# Socioeconomic, demographic and landscape factors associated with cutaneous leishmaniasis in Kurunegala District, Sri Lanka

**DOI:** 10.1186/s13071-020-04122-1

**Published:** 2020-05-12

**Authors:** Tharaka Wijerathna, Nayana Gunathilaka, Kithsiri Gunawardena, Wasana Rodrigo

**Affiliations:** 1 Department of Parasitology, Faculty of Medicine, University of Kelaniya, Ragama, Sri Lanka; 2 Biotechnology Unit, Industrial Technology Institute, Colombo 07, Sri Lanka

**Keywords:** Leishmaniasis, Sand fly, Risk factors, Socioeconomic

## Abstract

**Background:**

Leishmaniasis is a neglected tropical disease that affects countries in the developing world. In Sri Lanka, cutaneous leishmaniasis is the most common form of the disease. It is prevalent in dry and intermediate zones, mostly associated with rural settings. Understanding basic risk factors is critical in the management of the disease with effective interventions. This study is focused on assessing the demographic, socioeconomic and landscape factors associated with leishmaniasis in Kurunegala District, Sri Lanka.

**Methods:**

A descriptive cross-sectional study was conducted. Households of the past patients and randomly selected households, which had no history of leishmaniasis cases were interviewed. The clinical, socioeconomic, demographic, landscape and awareness-related data were obtained using a pre-tested, interviewer-administered questionnaire.

**Results:**

A total of 101 patients and a similar number of controls were included in the study. All the patients had the cutaneous form of the disease. Housewives and personnel with monthly incomes less than Rs. 10,000 (56.76 USD) were 3.9- and 9.5-times more prone to the disease, respectively, according to multivariate analysis. Presence of decaying garbage, termite hills, unclear areas, wet soil and gardening areas were always associated with the increased odds of acquiring the disease.

**Conclusions:**

Demographic factors do not play a pivotal role in the prevalence of leishmaniasis in the area. Housewives, inhabitants with low incomes and individuals who live in areas with conditions suitable for sand fly breeding and resting are major groups with a higher risk of infection. Special attention must be given in raising awareness and environmental management in control activities.
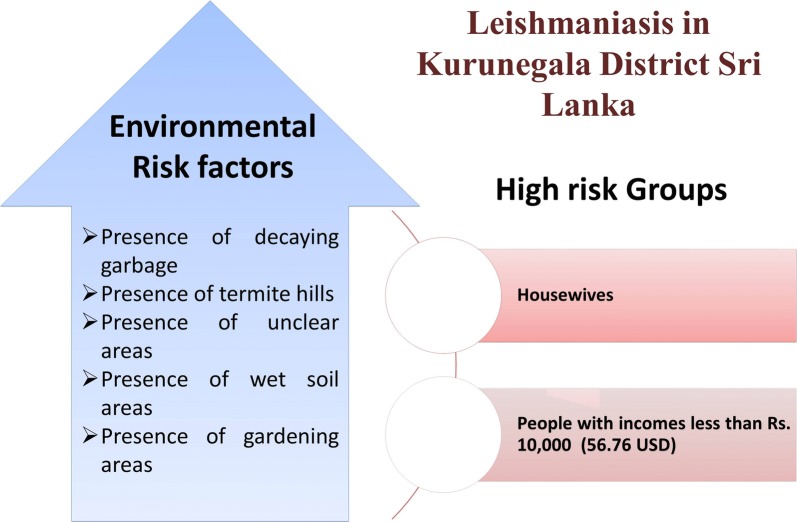

## Background

Leishmaniasis is a neglected tropical disease that mostly affects countries in the developing world [1]. Cutaneous leishmaniasis (CL) is among the main clinical manifestations of this disease with 0.6–1.0 million cases per year worldwide [1]. In Sri Lanka, CL is the most common type of leishmaniasis. Most patients have been recorded from dry and intermediate zones (transition zone between the dry and wet zones) [2, 3] until an outbreak was observed from the wet zone in 2018 [4].

In Sri Lanka, leishmaniasis is caused by *Leishmania donovani* zymodeme MON-37 [5] and transmitted by *Phlebotomus argentipes* [6]. *Phlebotomus argentipes* prefers areas with wet and humid soil enriched with decaying organic matter as breeding sites [7]. Dark and disorganized areas such as storerooms, piles of reed mats and storage areas of old and broken material are the main potential indoor diurnal resting places of sand flies in these areas [8]. The betel and pepper cultivated fields and the inner side of termite mounds can be considered as main outdoor diurnal resting places [8]. Leishmaniasis in Sri Lanka, which is caused by *L. donovani* is most likely to be an anthroponosis. However, few published studies indicate the possibility of dogs being a reservoir host although there is no sufficient evidence [9, 10].

Risk factors of leishmaniasis include poor household characteristics, low level of education, lack of cleanliness in the surrounding environment and poor awareness about the disease [11–14]. The housing conditions such as cracked walls, dark humid corners, damp floors, and mud-plastered walls, which permit the easy entry, resting and breeding of sand flies, are known to increases the risk of infection of the inhabitants [14–17]. Furthermore, poor awareness of vectors, vector behaviour and protective measures of the disease, which is partly a result of the poor level of education, are also associated with a high risk of acquiring the infection [11–14]. Poor sanitization and garbage collection in the surroundings, which result in an environment preferable for sand fly survival, are also reported as risk factors for leishmaniasis [11, 18, 19]. Gender inequality is often encountered concerning leishmaniasis. Most often males are more prone to the disease than females [20–22]. However, this is not consistent across studies. Some studies suggest similar effects for both sexes while some studies indicate a higher susceptibility of females [23, 24]. Children usually have a lower risk than adults to acquire the infection [25, 26]; however, this is also not consistent for all settings. Other studies suggest that children have a higher risk [27].

Some associated factors are limited to a specific form of the disease [21–24, 28]. Further, these risk factors may differ from one geographical region to another [21, 22, 24, 28]. Therefore, demography, epidemiology of the disease and risk factors at endemic setting should be studied to determine proper interventions. Hence, this study was conducted to understand major demographic, socioeconomic and landscape factors associated with leishmaniasis in Kurunegala district, which is one of the highest disease-endemic districts in Sri Lanka.

## Methods

### Study area

Kurunagala district (7°45’ N, 80° 15’ E) is located in the North-Western Province of Sri Lanka covering 4812.7 km^2^. Approximately 1,676,000 inhabitants live in the area within nearly 439,065 households [29]. About 32.6% of the population depends on agriculture-related employment while 36.6% are involved in other non-agricultural services followed by industry [30]. The prevalence of the disease in Sri Lanka is approximately 0.017%. Kurunegala is one of the endemic districts with high prevalence of CL. According to patient records from 2009 to 2016, there was nearly an 18-fold increase in the leishmaniasis incidence in this area. In 2018, a total of 533 cases were reported with an approximate prevalence of 0.37% [4].

### Selection of the study site

**Fig. 1:**
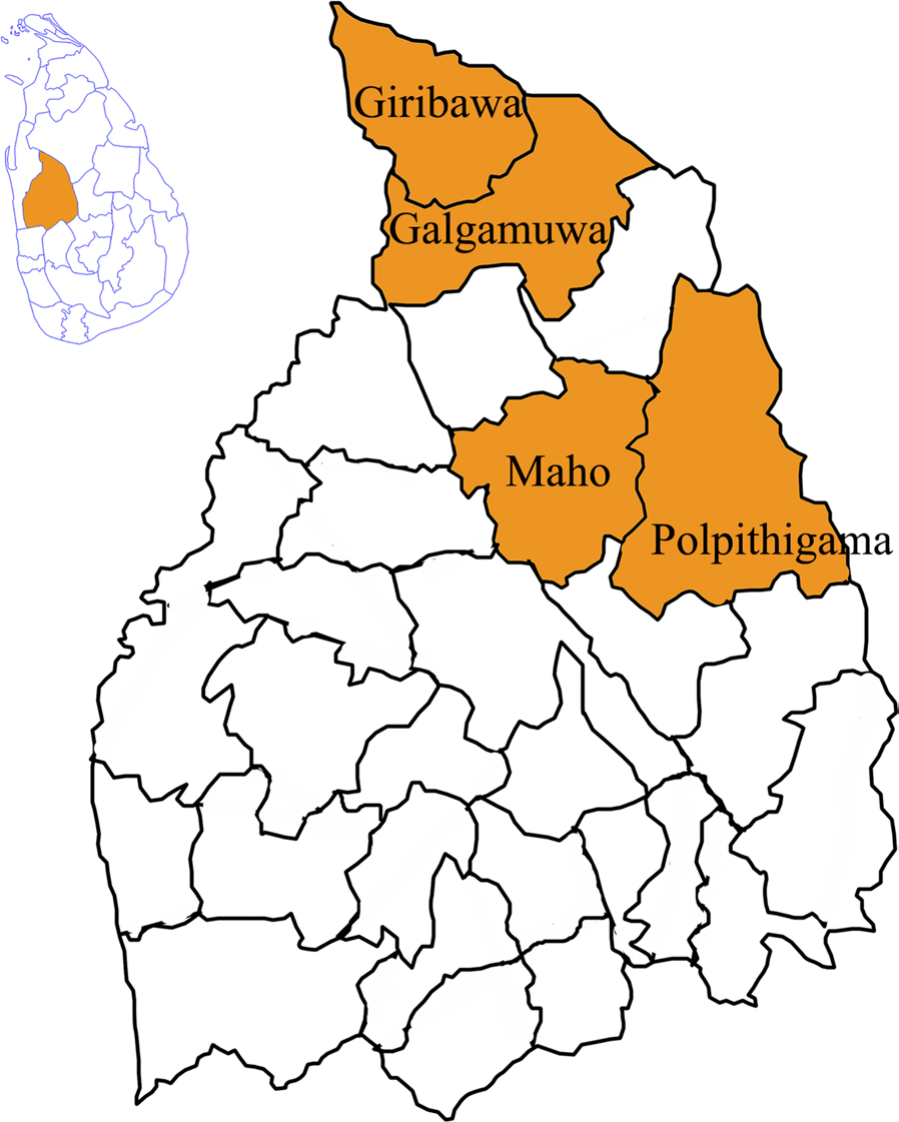
The location of selected MOH areas within an extracted map of Kurunegala District, Sri Lanka

The district of Kurunegala has 26 Medical Officer of Health (MOH) areas. The prevalence of leishmaniasis in all MOH areas is not uniform. The disease prevalence is considerably higher in some MOH areas. Galamuwa, Giribawa Maho and Polpithigama MOH areas, which are on the list of high disease occurrences, were selected for the present study (Fig. 1).

For the case group, all patients in four selected areas during the period under study (2013–2016) were included. For the control group, a list of villages with previous records of patients was prepared and arranged alphabetically. This list was numbered consecutively, and villages were selected using a random number table. The selected villages were visited, and every third household was considered for the interview. If there were no records of a household member having CL, this household was included into the control group. This process was continued until the required sample size (*n* = 101 matched with 101 cases, case:control = 1:1) was achieved [31].

### Data collection

Records of the leishmaniasis patients who reside in Galgamuwa, Giribawa, Maho and Polpithigama MOH areas that notified the relevant MOH office during 2013–2016 were obtained. Patient households were visited, and a pre-tested structured questionnaire was administered. If the patient was below 18 years-old, the parent or guardian was interviewed.

**Fig. 2:**
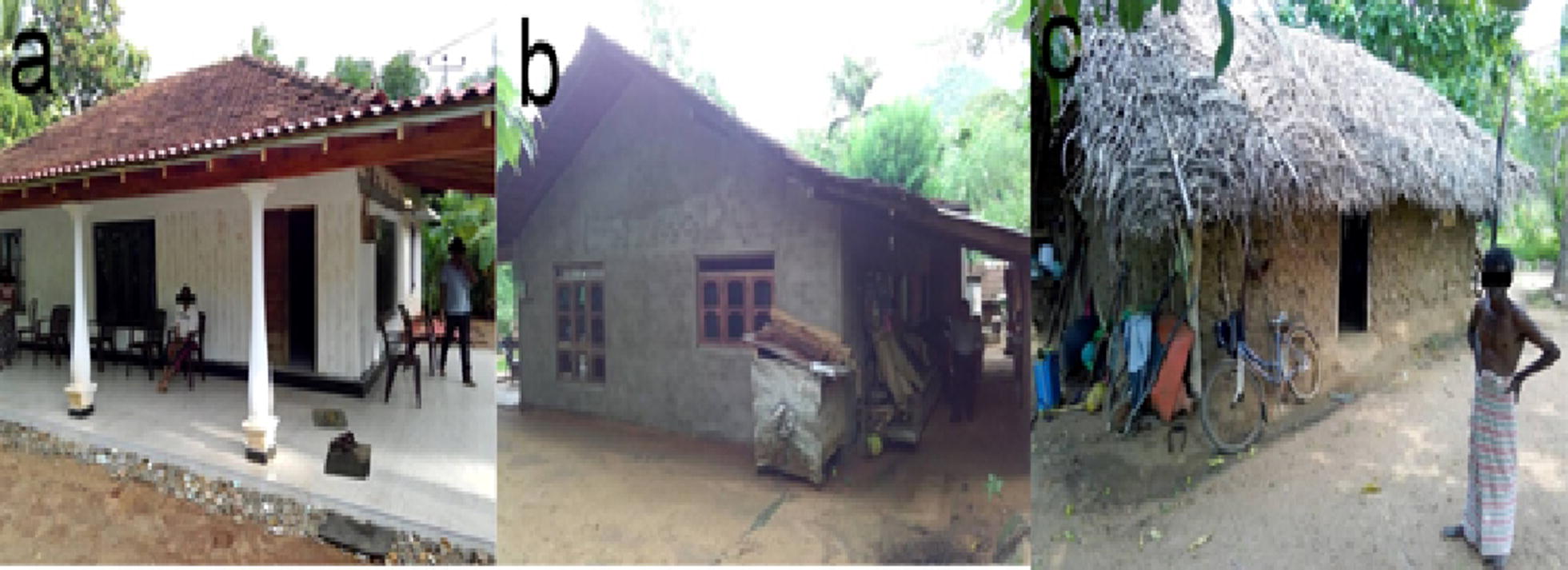
Examples for house types. **a** Good (plastered cement walls with tiled or asbestos roofs). **b** Moderate (un-plastered brick walls with tiled or asbestos roofs). **c** Poor (clay walls, thatched, or aluminum sheet roof)

The basic demographic information such as age in years, level of education, marital status, gender and the family size of the respondent was recorded. Socioeconomic factors such as occupation, monthly income and house conditions were recorded. House conditions were categorized as “poor”, “moderate” and “good” based on roof, wall and floor characteristics [32]. The houses having plastered cement walls with tiled or asbestos roofs were categorized as “good” (Fig. 2a) while un-plastered brick walls with tiled or asbestos roofs were considered as “moderate” (Fig. 2b). All other types of housing were grouped as “poor” (Fig. 2c).

The surroundings of the households were observed and landscape characteristics such as the presence of decaying garbage, termite hills, manna bushes, water streams, unclear areas (dark areas with trees or/and shrubs) and gardening areas within approximately a 100 m radius were recorded. These factors were selected based on the published literature and preliminary studies conducted in these areas [12, 33, 34]. In the awareness assessment, a series of questions were asked providing choices where applicable. However, guesses were not included as a response. In the recording of clinical features, clinical records available with the patient were referred to for the confirmation of responses given by patients.

### Data analysis

Collected data were entered into EpiData version 4.4.3.1 (https://www.epidata.dk) data handling software package. These data were exported as CSV files and analyzed in IBM SPSS Statistics for Windows, Version 25.0 (IBM Corp., Armonk, NY, USA). The demographic, landscape and socioeconomic factors associated with CL in the case and control groups were compared by the Chi-square test for independence. Odds ratios were calculated with a 95% confidence interval for each significantly different variable through univariate and multivariate regression analysis. For each categorical variable, the last category was selected as the reference category to calculate the odds ratio. The backward conditional logistic regression model was used for univariate analysis. The number of patients was selected as the dependent variable and the selected risk factor was considered as the independent variable. Nagelkerke *R*^2^ value and the significance of the odds ratio were considered for the interpretation of univariate analysis results. For multivariate analysis, the multinomial logistic regression model was used. Age and gender were included as covariates to exclude the confounding effects. The number of patients was considered as the dependent variable and the selected risk factor was considered as the independent variable. The likelyhood ratio test and the goodness-of-fit-test were performed to assess the model.

## Results

**Fig. 3:**
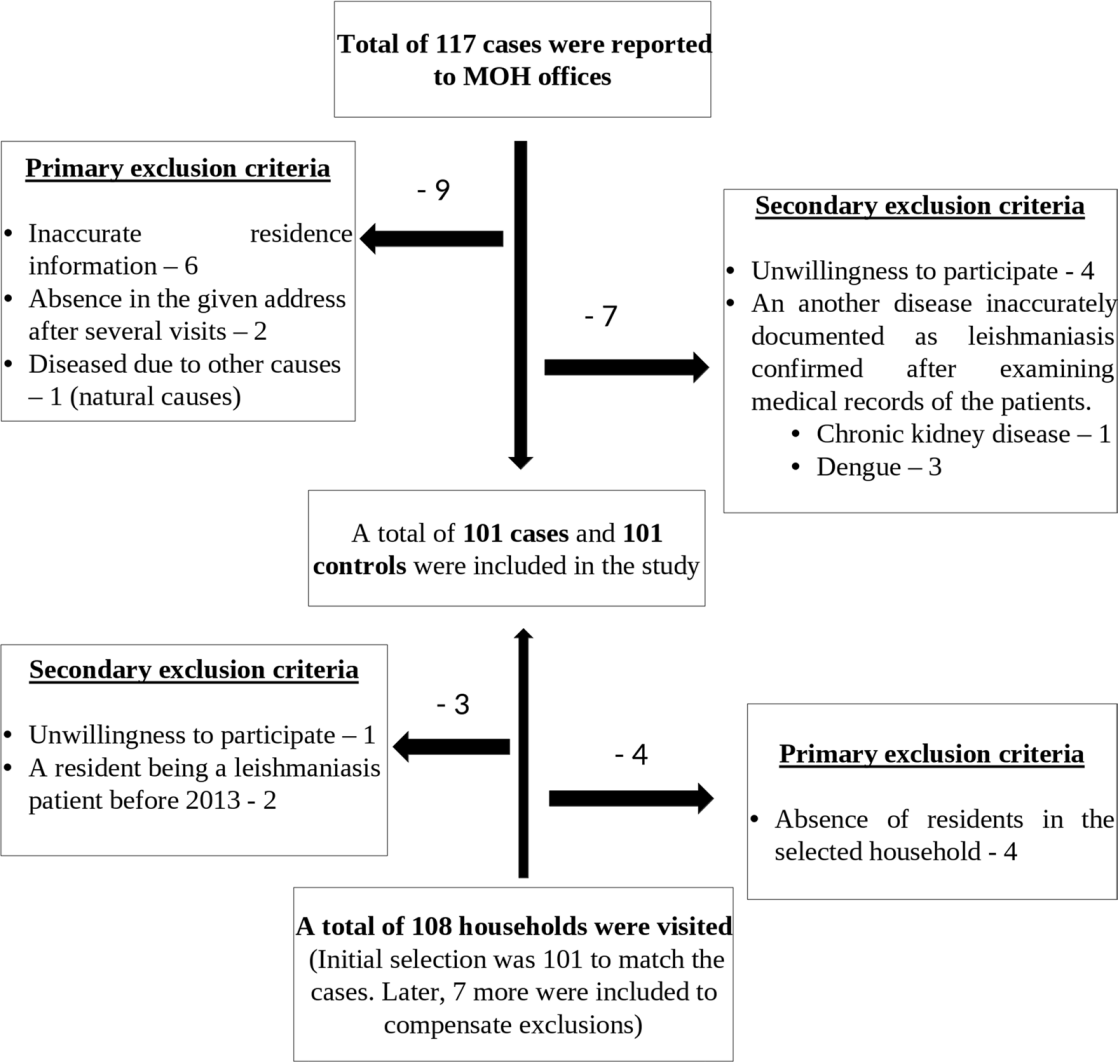
Inclusion and exclusion criteria of the cutaneous leishmaniasis cases and controls in the household survey in Galgamuwa, Giribawa, Maho and Polpithigama MOH areas in Kurunegala District, Sri Lanka

A total of 117 patients had been reported from all four MOH areas during 2013–2016. Among them, a total of 101 patients were included in the study. Other patients were excluded due to one or more of the reasons; inaccurate contact information provided by the patients, absence at the provided address after 3 visits and change of residency to a different area, false-positive reports as confirmed after referring to medical records and unwillingness to participate (Fig. 3). The spatial distribution of cases and controls is illustrated in Fig. 3. The numbers of patients from each area are tabulated in the Additional file 1: Table S1.

### Clinical features

**Fig. 4:**
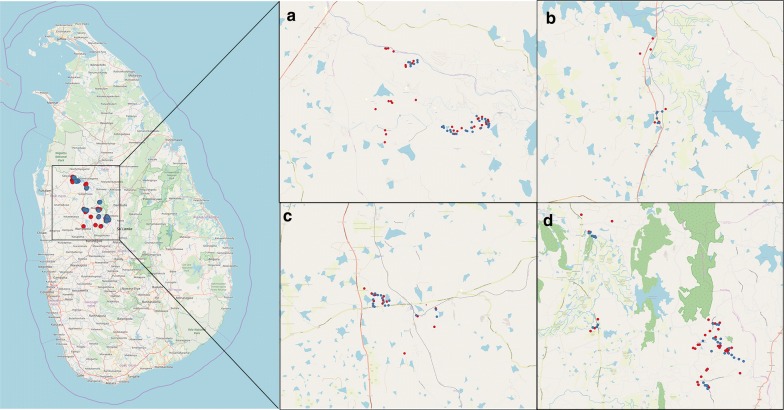
Distribution of cutaneous leishmaniasis cases and controls in a map of Sri Lanka (1:4,000,000) and each MOH area (1:80,000): **a** Giribawa, **b** Galgamuwa, **c** Maho, **d** Polpithigama. Red dots indicate the locations of the patients while the blue dots indicate the location of controls

All patients had the cutaneous form of the disease and none of the patients had visceral leishmaniasis or mucocutaneous leishmaniasis symptoms. The most common type of the lesion encountered was nodules (*n* = 64, 63.36%) followed by ulcers (*n* = 31; 30.70%) and plaques (*n* = 6, 5.94%). Having more than one lesion was rare and observed only in 12 patients. The majority of the lesions were less than 15 mm in diameter (*n* = 72, 63.16%). The remainder were between 15–30 mm (*n* = 34, 29.82%) and rarely exceeded 30 mm (*n* = 8, 7.02%). The lesions were more prominent on the hands (*n* = 39, 34.21%) and face (*n* = 37, 32.45%) (Table 1). Only one patient had a recurrent infection two months after completion of the treatment episode (Fig. 4).

**Table 1:** Clinical features of patients confirmed for cutaneous leishmaniasis

Feature		No. of patients (%)
Lesion type		
Ulcers		31 (30.69)
Nodule		64 (63.37)
Plaque		6 (5.94)
Number of lesions		
1		89 (88.12)
2		11 (10.89)
3		1 (0.99)
Lesion size (mm)		
< 15		72 (63.16)
15-30		34 (29.82)
> 30		8 (7.92)
Lesion location		
Face		37 (32.46)
Earlobe		9 (7.89)
Torso		14 (12.28)
Legs		15 (13.15)
Hands		39 (34.22)

### Demographic features

Most of the patients were male (*n* = 56, 55.44%) within the age group of 26–35 years-old (*n* = 23, 22.77%) which covers the working-age (Table 2). The majority of the patients were married (*n* = 84, 83.17%) with an average of 1–3 members in the family (*n* = 58, 57.42%). Seventy-five patients (67.32%) had received education at least up to the General Certificate of Education in Ordinary Level (GCE O/L). The Chi-square test indicated that the two groups (case and control) significantly differed with respect to age (*P *< 0.05), gender (*P *< 0.001) and marital status (*P *< 0.05). The size of the family and the level of education did not differ significantly among the two groups (*P *> 0.05) (Table 2).

**Table 2:** Results for the comparison of demographic, socioeconomic and landscape factors between the case and control groups by Chi-square test

Factor		Case *n* (%)	Control *n* (%)	*χ*^2^	*P*-value
Age (years)					
< 15		9 (8.91)	1 (0.99)	24.690	0.001
15–25		8 (7.92)	5 (4.95)		
26–35		23 (22.77)	51 (50.49)		
36–45		22 (21.78)	21 (20.79)		
46–55		18 (17.82)	13 (12.87)		
56–65		10 (9.90)	7 (6.93)		
66–75		8 (7.92)	1 (0.99)		
76–85		3 (2.97)	2(1.98)		
Gender					
Male		56 (55.45)	82 (81.19)	15.461	< 0.001
Female		45 (44.55)	19 (18.81)		
Marital status					
Married		84 (83.17)	94 (93.07)	4.728	0.030
Unmarried		17 (16.83)	7 (6.93)		
Family size					
1–3		58 (57.43)	49 (48.51)	1.668	0.434
4–6		41 (40.59)	49 (48.51)		
> 6		2 (1.98)	3 (2.98)		
Education					
Grade 1–4		6 (5.94)	2 (1.98)	4.259	0.642
Grade 5		23 (22.77)	27 (26.73)		
O/L		46 (45.54)	53 (52.48)		
A/L		20 (19.80)	16 (15.84)		
Higher education		2 (1.98	1 (0.99)		
None		4 (3.96)	2 (1.98)		
Major occupation					
Armed forces		3 (2.98)	6 (5.94)	24.429	0.005
Transport service worker (driver/conductor)		6 (5.94)	4 (3.96)		
Farmer		33 (32.67)	29 (28.71)		
Government server		5 (4.95)	9 (8.91)		
Labourer		2 (1.98)	3 (2.98)		
Mason		6 (5.94)	16 (15.84)		
Private sector employee		3 (2.98)	7 (6.93)		
Self employed		8 (7.92)	13 (12.87)		
Housewife		29 (28.71)	8 (7.92)		
Student		6 (5.94)	6 (5.94)		
Monthly income Rs. (USD)					
< 10,000 (< 56.76)		20 (19.80)	3 (2.98)	15.372	0.002
10,001–20,000 (56.76–113.52)		33 (32.67)	41 (40.59)		
20,001–30,000 (113.52–170.28)		27 (26.73)	26 (25.74)		
> 30,000 (> 170.28)		21 (20.79)	31 (30.69)		
House condition					
Poor		11 (10.89)	13 (12.87)	2.745	0.253
Moderate		52 (51.49)	61 (60.40)		
Good		38 (37.62)	27 (26.73)		
Decaying garbage					
Present		57 (56.44)	25 (24.75)	20.559	<0.001
Absent		44 (43.56)	76 (75.25)		
Termite hills					
Present		45 (44.55)	34 (33.66)	9.272	<0.05
Absent		56 (55.45)	67 (66.34)		
Manna bushes					
Present		50 (49.50)	43 (42.57)	0.741	0.389
Absent		51 (50.50)	58 (57.43)		
Water streams					
Present		47 (46.53)	60 (60.40)	3.358	0.067
Absent		54 (53.47)	41 (39.60)		
Unclear areas					
Present		63 (62.38)	40 (39.60)	10.070	< 0.05
Absent		38 (37.62)	61 (60.40)		
Areas with wet soil					
Present		52 (51.49)	30 (29.70)	9.936	< 0.05
Absent		49 (48.51)	71 (70.30)		
Gardening areas					
Present		50 (49.50)	26 (25.74)	11.806	0.001
Absent		51 (50.50)	75 (74.26)		
Animals owned or frequently visiting the home harden and household					
Dogs		77 (76.24)	51 (50.50)	8.891	0.180
Cats		36 (35.64)	17 (16.83)		
Cattle		8 (7.92)	8 (7.92)		
Chicken		2 (1.98)	4 (3.96)		
Wild boar		12 (11.88)	4 (3.96)		
Monkeys		2 (1.98)	0 (0)		
Rodents		13 (12.87)	3 (2.98)		
Heard of the disease? (before the infection)					
Yes		25 (24.75)	66 (65.35)	33.617	< 0.001
No		76 (75.25)	35 (34.65)		
If yes, source					
TV/radio		3 (12.00)	8 (12.12)	36.816	< 0.001
Printed media		1 (4.00)	2 (3.03)		
Friend		19 (76.00)	39 (59.09)		
Health worker		2 (8.00)	17 (25.75)		
Seen a patient? (before infection)					
Yes		16 (15.84)	43 (42.57)	17.454	< 0.001
No		85 (84.16)	58 (57.43)		
If yes, source?					
Village		15 (93.75)	37 (86.05)	16.802	< 0.001
Outside village		1 (6.25)	4 (9.30)		
TV/printed media		0 (0.00)	2 (4.65)		
Causative organism					
Correct		2 (1.98)	0 (0)	2.020	0.155
Wrong		0 (0)	0 (0)		
Unaware		99 (98.02)	101 (100)		
Mode of transmission					
Correct		20 (19.80)	1 (0.99)	6.000	0.199
Wrong		3 (2.98)	8 (7.92)		
Unaware		78 (77.22)	92 (91.09)		
Method of diagnosis					
Correct		1 (0.99)	0 (0)	1.005	0.316
Unaware		100 (99.01)	101 (100)		
Peak biting time					
Correct		5 (4.95)	0 (0)	6.184	0.045
Wrong		1 (0.99)	0 (0)		
Unaware		95 (94.06)	101 (100)		
Protective measures					
Correct		5 (4.95)	2 (1.98)	2.451	0.294
Wrong		11 (10.89)	7 (6.93)		
Unaware		85 (84.16)	92 (91.09)		

*Note*: *P *< 0.05 indicates a statistically significant result

Univariate analysis comparing cases and controls showed that age does not increase or decrease the odds of having the disease at significant levels. However, children less than 15 years-old had higher odds of acquiring the disease (OR: 6.000, 95% CI: 0.390–92.277) than the other two groups, though not statistically significant (Table 3). Interestingly, males had reduced odds of becoming infected than females (OR: 0.273, 95% CI: 0.144–0.520). On the other hand, unmarried individuals were less likely to be leishmaniasis patients than married individuals (OR: 0.368, 95% CI: 0.145–0.931). Although associations indicated by odds ratios remained the same for gender, after the adjustment on age in the multivariable conditional logistic regression model, it was no longer statistically significant.

**Table 3:** Results of the univariate logistic regression analysis of the factors associated with the transmission of CL in Galgamuwa, Giribawa, Polpithigama and Maho MOH areas in the Kurunegala district, Sri Lanka

Variable	Case	Control	OR	95% CI	*R*^2^
Age (years)					
< 15	9	1	6.000	0.390–92.277	0.165
15–25	8	5	1.067	0.129–8.793	
26–35	23	51	0.204	0.047–1.923	
36–45	22	21	0.709	0.106–4.607	
46–55	18	13	0.935	0.134–6.335	
56–65	10	7	0.962	0.125–7.275	
66–75	8	1	0.232	0.343–82.827	
76–85	3	2	1		
Gender					
Male	56	82	0.273*	0.144–0.520	0.107
Female	45	19	1		
Marital status					
Married	84	94	0.368*	0.145–0.931	0.032
Unmarried	17	7	1		
Major occupation					
Armed forces	3	6	0.5	0.084–2.994	0.153
Transport service worker (driver/conductor)	6	4	1.499	0.274–8.196	
Farmer	33	29	1.138	0.330–3.922	
Government server	5	9	0.556	0.115–2.681	
Labourer	2	3	0.667	0.080–5.525	
Mason	6	16	0.375	0.086–1.631	
Private sector employee	2	7	0.286	0.041–1.980	
Self employed	8	13	0.615	0.147–2.584	
Housewife	29	8	3.623*	0.915–14.286	
Student	6	6	1		
Monthly household income in Rs. (USD)					
< 10,000 (< 56.76)	20	3	9.841*	2.593–37.357	0.107
10,001–20,000 (56.76–113.52)	33	41	1.188	0.579–2.439	
20,001–30,000 (113.52–170.28)	27	26	1.533	0.708–3.319	
> 30,000 (> 170.28)	21	31	1		
Decaying garbage					
Present	57	25	3.886*	2.134–7.079	0.132
Absent	44	76	1		
Termite hills					
Present	45	34	2.408*	1.361–4.261	0.061
Absent	56	67	1		
Unclear areas					
Present	63	40	2.487*	1.410–4.387	0.066
Absent	38	61	1		
Areas with wet soil					
Present	52	30	2.512*	1.409–4.478	0.065
Absent	49	71			
Gardening areas					
Present	50	26	2.790*	1.542–5.050	0.077
Absent	51	75			

*Statistically significant (*P *< 0.05)

*Abbreviations*: OR, odds ratio; *R*^2^, Nagelkerke *R*^2^ value

### Socioeconomic characteristics

Majority of the patients were farmers (*n* = 33, 32.67%), followed by housewives (*n* = 29, 28.71%). Most of the patients had a monthly income of Rs. 10,001–20,000 (56.76–113.52 USD) (*n* = 33, 32.67%) (Table 2). Patients predominantly occupied houses with moderate conditions (*n* = 52, 51.48%), followed by houses with good (*n* = 38, 37.62%) and poor (*n* = 11, 10.89%) conditions. Major occupations (*χ*^2^ = 24.429, *df* = 10, *P *< 0.05) and monthly income (*χ*^2^ = 15.372, *df* = 3, *P *< 0.05) were significantly different between case and control groups. However, the univariate conditional regression analysis suggested that being a housewife has elevated odds of becoming infected (OR: 3.623, 95% CI: 0.915–14.286). According to the multivariate analysis, housewives were 3.9 times more likely to become infected (95% CI: 0.606–12.658). The individuals with a monthly income of< Rs. 10,000 (56.76 USD) were 9.5 times more likely to become the infected than those who earn> Rs. 30,000 (170.28 USD) per month (Table 4).

**Table 4:** Results of the multinomial logistic regression of the factors associated with the transmission of CL in Galgamuwa, Giribawa, Polpithigama and Maho MOH areas in the Kurunegala district, Sri Lanka

Variable	Case	Control	OR	95% CI	LR test
Age (years)					
< 15	9	1	0.245	0.015–3.996	< 0.001
15–25	8	5	0.968	0.112–8.400	
26–35	23	51	3.957	0.588–26.640	
36–45	22	21	1.541	0.222–10.701	
46–55	18	13	1.165	0.162–8.386	
56–65	10	7	1.472	0.180–12.030	
66–75	8	1	0.261	0.016–4.306	
76–85	3	2	1		
Gender					
Male	56	82	0.273	0.144–0.521	< 0.001
Female	45	19	1		
Marital status					
Married	84	94	0.350	0.122–1.001	< 0.001
Unmarried	17	7	1		
Major occupation					
Armed forces	3	6	0.756	0.117–4.878	0.001
Transport service worker (driver/conductor)	6	4	2.278	0.380–13.700	
Farmer	33	29	1.441	0.368–5.650	
Government server	5	9	0.585	0.111–3.067	
Labourer	2	3	1.016	0.112–9.259	
Mason	6	16	0.516	0.107–2.481	
Private sector employee	2	7	0.297	0.040–2.198	
Self employed	8	13	0.818	0.170–3.940	
Housewife	29	8	3.937*	0.606–12.658	
Student	6	6	1		
Monthly household income in Rs. (USD)					
< 10,000 (< 56.76)	20	3	9.524*	2.439–37.037	< 0.001
10,001–20,000 (56.76–113.52)	33	41	1.048	0.493–2.232	
20,001–30,000 (113.52–170.28)	27	26	1.260	0.559–2.841	
> 30,000 (> 170.28)	21	31	1		
Decaying garbage					
Present	57	25	4.131*	2.188–7.153	< 0.001
Absent	44	76			
Termite hills					
Present	45	34	2.427*	1.337–4.407	0.003
Absent	56	67			
Unclear areas					
Present	63	40	2.742*	1.502–5.005	0.001
Absent	38	61	1		
Areas with wet soil					
Present	52	30	2.492*	1.360–4.564	0.003
Absent	49	71	1		
Gardening areas					
Present	50	26	2.577*	1.389–4.779	0.002
Absent	51	75	1		

*Statistically significant (*P *< 0.05)

*Abbreviations*: OR, odds ratio; LR, likelihood ratio

### Landscape factors

The presence of decaying garbage in the surroundings was observed amongst the majority of patient households (*n* = 57, 56.44%). Moreover, many of the patient households (*n* = 63, 62.38%) had uncleared surroundings with pepper cultivations, banana plant aggregations and vegetation that provide shady and dark conditions. The number of patient households that had nearby wet soil areas was slightly higher (*n* = 52, 51.49%). The main wet soil areas that were noted were the soil in open pits where collected water, turned soil in Chena cultivations, the soil surrounding cemented water tanks, the soil at the base of the cultivated plants which are regularly watered, paddy fields, and the ground surrounding animal huts. Among the landscape factors, presence or absence of decaying garbage, termite hills, unclear areas, areas with wet soil and gardening areas differed significantly between the case and control groups (*P *< 0.05). All these five factors were associated with increasing the risk of infection, according to the univariate analysis (Table 3). Multivariate logistic regression analysis indicated that the presence of decaying garbage increases the risk of infection by 4 times. The presence of termite hills, unclear areas, areas with wet soil and gardening areas increases the odds of having the disease by 2.4, 2.7, 2.4 and 2.5 times, respectively.

Ownership of animals or animals which frequently visit the garden at home did not show a significant difference between the case and control groups (*χ*^2^ = 8.891, *df* = 6, *P* = 0.180). However, the majority of the patients (*n* = 77, 76.24%) had pet or feral dogs in the surrounding area. Cats, cattle, chickens, wild boars, monkeys and rodents (rats, shrews and squirrels) were the other commonly visiting animals to home gardens in these areas.

### Awareness about the disease

The majority of the patients (*n* = 76, 75.25%) had not heard about a disease called leishmaniasis before infection, while most of the control group (*n* = 66, 65.35%) had heard about the disease. The majority had heard about the disease from a friend, and this was true for both case (*n* = 19) and control groups (*n* = 39). Awareness from health workers (*n* = 17, 16.83%) was another source of information in the control group. Only 16 (15.84%) individuals had seen someone else with the infection before they became infected (Table 2). Only two individuals who had the infection knew that the disease was caused by a protozoan parasite. No one in the control group was aware of the parasite.

A total of 20 patients mentioned that the mode of transmission is by sand flies. Only one person from the control group marked the correct answer that leishmaniasis is transmitted by the sand fly *Phlebotomus argentipes*. No one was aware of any diagnostic methods of leishmaniasis, except one patient who was aware of microscopy. Only five individuals in the case group correctly answered the question for peak biting time. Very few in the case (*n* = 5) and control groups (*n* = 2) were aware of the correct protective measures to prevent vector biting, other individuals failed to reply. No significant difference between case and control groups was observed regarding knowledge about the disease, except for the peak biting time, which was higher for individuals who had had the disease previously. However, hearing about the disease and seeing a patient with the disease was significantly different between the two groups, where only very few from the case group had heard about the disease (*n* = 25) or had seen a patient before they acquired the infection (*n* = 16).

## Discussion

The South Asian region has suffered from the devastating epidemics of leishmaniasis since the early 1980s [35]. Nonetheless, substantial progress has been made in controlling leishmaniasis in this region through successful diagnosis *via* active detection, complete case management, effective vector control measures, community mobilization and operational use of research generated knowledge [36, 37]. In Sri Lanka, leishmaniasis has received little attention from the authorities despite regular increases in the number of cases every year since 2011 [38]. One of the main limitations in the control of this disease in Sri Lanka is the lack of research at the regional level that would highlight the important risk factors that need to be addressed through control programmes. In this regard, the present study revealed valuable information.

The demographic factors associated with CL differ regionally, in some areas the elderly population is mostly affected [39], or in other areas the younger population [40]. The younger population was the most affected group according to the univariate analysis. However, this did not significantly predict the disease incidence. Although gender and marital status showed statistically significant associations according to the univariate analysis, it did not significantly predict the disease incidence as indicated by the adjusted odds ratios. Hence, the observed significant relationships in the univariate analysis are likely to be a result of confounding effects [41]. Therefore, demographic factors may not significantly affect the odds of having the disease.

In general, outdoor occupations increase the risk of leishmaniasis incidence due to a greater risk of being bitten by sand flies [12, 42, 43]. Furthermore, the results of previous studies in Sri Lanka have indicated that *P. argentipes* is more likely to transmit the parasite to humans as outdoor bites [6]. As observed in the present study and previous studies [27, 40, 44], housewives are at risk of receiving infection. This may be due to their outside routine activities such as picking firewood, cleaning home gardens, washing clothes and bathing at outdoor water sources. Furthermore, women of farming families often denominate themselves as housewives, but they tend to assist their husbands in farming activities, which make them equally prone to vector sand fly bites. Therefore, this study indicates the importance of more extensive studies to assess the effects of day to day activities on the acquisition of this infection.

Leishmaniasis is considered a neglected tropical disease, where the poorest of the population is affected [45]. This has been demonstrated in the present study, as the odds of infection with leishmaniasis increased with poverty. Those who had a monthly income of< Rs. 10,000 (56.76 USD) were potentially at risk of infection 9.5 times higher compared to those who had a monthly income of> Rs. 30, 000 (170.27 USD). Generally, neglected tropical diseases affect poor populations due to poor sanitation, poor housing conditions, and lack of access to essential nutrition [46]. In this case, housing condition was not a significant factor in the both case and control groups. Therefore, in these study areas, house condition can be excluded as a major risk factor for the disease. However, with this knowledge, further studies would provide a better insight into other associated socioeconomical risk factors.

In any vector-borne disease, the presence of potential breeding/resting places of the vectors and reservoirs are associated with an increase in disease incidence [28, 46, 47]. In the present study, presence of potential breeding places for sand flies (decaying garbage, termite hills and areas with wet soil) and potential adult resting places (gardening areas and unclear areas) were associated with increased odds of leishmaniasis incidence. This is consistent with previous studies which indicated a higher risk of acquiring leishmaniasis with the presence of suitable resting and breeding sites in close proximity [11, 47]. Although reservoir hosts of the parasite in Sri Lanka is unclear, dogs are suspected to be a reservoir host [9, 10]. Nevertheless, the presence or absence of dogs, other pets (cats), livestock (cattle and chicken), stray animals (wild boar, rodents and monkeys) commonly visiting home gardens did not differ significantly between the two groups (*P *> 0.05). The same scenario has been observed from another study conducted in a different region of Sri Lanka [11]. The absence of increased exposure to potential reservoirs by patients is not adequate in making a strong conclusion about the role of reservoir hosts of the disease. The parasite *L. donovani* is known to have an anthroponotic transmission cycle, according to studies from other countries [48–50]. A study suggested the possibility of cattle being a reservoir host for *L. donovani* in Bangladesh [51]. However, more evidence indicates that the transmission may not involve an animal reservoir [48–50]. In align with these findings, studies from Sri Lanka show no infections in rodents and low infection rates in dogs [9, 10]. Therefore, it is more likely that the parasite has an anthroponotic transmission cycle in this area of Sri Lanka. However, further studies on the interactions between the reservoir host, vector, parasite and human hosts are essential for a better understanding of the involvement of a reservoir in disease transmission.

Awareness about the disease in both control and case groups was very poor. No one without a history of leishmaniasis infection knew about the causative organism, while only two individuals knew that the disease is caused by a parasite. Some of the infected individuals mentioned the name of the vector correctly. But, none of them were aware what kind of insect it was. When they were shown a live specimen, they identified it by another name other than the Sinhalese term coined by the scientific community. Villagers used the same Sinhalese term for both biting midges and sand flies (“Ho haputta”). Researchers used the exact Sinhalese translation of the term sand fly (“Weli massa”) to introduce sand flies. This often confused the villagers, as it is not a name which is commonly known among the community. The Sinhala term for sand flea (“Weli makka”) is very similar to the Sinhala term used for sand flies. Therefore, inhabitants in these disease-endemic areas often misinterpret sand flea as the vector of leishmaniasis. Hence, the majority of the incorrect responses during the study was sand flea as the disease vector. In two cases, inhabitants believed that the disease can be transmitted by the fruit fly and the lesion is due to the eggs laid by the fruit fly on the skin. This assumption, which they believed to be true, was based on their experience in vegetable and fruit cultivations. Knowledge of protective measures was also very poor among the study population. Only a few (*n* = 7) respondents were aware of the correct protective measures such as wearing protective clothing, the use of insect repellents and minimizing outdoor activities during dusk/dawn. One of the most common responses was the use of mosquito nets, which was considered as a wrong response, as sand flies can crawl through the mesh of mosquito nets [52, 53]. Another incorrect response was staying away from sand to avoid sand fly biting, and thereby to prevent from the infection.

In statistical analyses, the two groups were initially compared using the Chi-square test. Statistically significant values were assessed by univariate logistic regression. However, the results may be affected by confounding factors. Therefore, the odds ratios adjusted for age and gender were calculated by multivariate logistic regression. However, certain factors showed significant differences between the two groups according to the Chi-square test but did not show a relationship once adjusted for age and gender. Therefore, initially existing relationships could be attributed to the confounding effects.

During the survey, the respondents were asked to recall the time before acquiring infection. This may have resulted in inaccuracies of the information provided. Furthermore, patients may not always provide correct answers about their education level and income due to social stigma. Another caveat of this study is that we recorded only the chief occupation of the patients. Most of the inhabitants were involved in agriculture-related activities in addition to their main occupation. Therefore, monitoring of their day to day activities, other than the main occupation is required. Furthermore, depending on the number of inhabitants in the study area, the ideal sample size for the study would be above 383 (at the 95% confidence level) [54]. Therefore, having only 101 patients for the study period is another limitation of this study. However, despite these unavoidable limitations, this study addresses some important aspects of the perception of the disease among at-risk communities. Therefore, the findings would be useful in implementing control programmes through a systematized action plan under the Ministry of Health, which has been identified as of paramount importance at present.

## Conclusions

This study demonstrates that the occupation, monthly income, and the presence of potential breeding and resting places in the environment are the main risk factors for the disease transmission in these areas. Among them, housewives, low-income individuals, inhabitants of households with decaying garbage, termite hills, unclear areas, areas with wet soil and gardening areas in the surrounding are at higher risk. Further, poor awareness about the disease among the general public is also a major negative point. Hence, the control efforts must be focused on raising awareness about protective measures, other important aspects and improvement of environmental/landscape conditions while implementing major control activities such as vector control, reservoir host control and effective case management.

## Supplementary information

**Additional file 1: Table S1.** The number of patients responded to the survey as the case group along with the percentage respondents of the total recorded patients identified from each Medical Officer of Health (MOH) area.

## Data Availability

Data supporting the conclusions of this article are included within the article and its additional file. A coded version of the dataset generated during the study will be available from the corresponding author upon reasonable request.

## Abbreviations

CL: cutaneous leishmaniasis
MOH: Medical Officer of Health
USD: United States dollar
