# Supplementary material for: Socioeconomic, demographic and landscape factors associated with cutaneous leishmaniasis in Kurunegala District, Sri Lanka
Source: Parasit Vectors. 2020 May 12;13:244. doi: 10.1186/s13071-020-04122-1 (PMC7216469; doi:10.1186/s13071-020-04122-1)
Supplement: Supplementary file 1 — Additional file 1: Table S1. The number of patients responded to the survey as the case group along with the percentage respondents of the total recorded patients identified from each Medical Officer of Health (MOH) area. [file 13071_2020_4122_MOESM1_ESM.docx]

**Additional file 1: Table S1.** The number of patients responded to the survey as the case group along with the percentage respondents of the total recorded patients identified from each Medical Officer of Health (MOH) area.

| MOH area | Number of respondents (%) |
| --- | --- |
| Polpithigama | 44 (43.56%) |
| Giribawa | 34 (33.67%) |
| Maho | 17 (16.83%) |
| Galgamuwa | 6 (5.94%) |
